# The Effect of Topical Vitamin A and E on Ischemic Random Skin Flap Survival

**DOI:** 10.29252/wjps.8.1.58

**Published:** 2019-01

**Authors:** Seyed Esmail Hassanpour, Khalil Rostami, Eznollah Azargashb, Kourosh Saberi, Seyyed Hosein Hamraz, Fatemeh Farajzadeh Vajari, Hojjat Molaei

**Affiliations:** 1 Department of Plastic and Reconstructive Surgery, Medicine School, Shahid Beheshti University of Medical Sciences, Tehran, Iran;; 2 Department of Plastic and Reconstructive Surgery, Medicine School, Semnan University of Medical Sciences, Semnan, Iran;; 3 Gynecologist surgeon, Private Practice, Tehran, Iran;; 4 Department of Plastic and Reconstructive Surgery, Imam Khomeini Hospital, Medicine School, Tehran University of Medical Sciences, Tehran, Iran

**Keywords:** Vitamin A, Vitamin E, Ischemia, Skin flap, Survival, Rat

## Abstract

**BACKGROUND:**

Ischemia of skin flaps is an important complication in reconstructive surgery. This study evaluated the efficacy of topical vitamins A and E on improving flap survival.

**METHODS:**

Twenty-four white-albino male rats were randomly divided into two groups of treatment and control. Standard rectangular, distally based dorsal random pattern skin flap was elevated. Intra-peritoneal cephazoline was administered to prevent any unexpected infection. No pharmaceutical agent was administered for the control group, but pure vaseline ointment. In treatment group, vaseline plus vitamins A and E were administrated daily after surgery for 10 days. The rats were evaluated on the 10^th^ day after surgery for viable and necrotic portions of the flaps.

**RESULTS:**

The mean values of necrosis in the flaps were 625±189.56 and 920.00±247.31 in the treatment and control groups, respectively. Vaseline plus vitamins increased flap survival significantly.

**CONCLUSION:**

Topical vitamins A and E may be effective pharmaceutical agents to increase viability of random skin flaps in rats. They can be added to vasoactive topical agents to reach better results.

## INTRODUCTION

Improvements of reconstructive fields such as burns, congenital deformities, tumor resections, trauma sequels and even aesthetic issues enhanced expectations. Reconstructive surgery as a remarkable life boat, uses flaps as its work horses. So random skin flap has been used for many years with acceptable outcomes. Despite tremendous progress in the past decades, flap surgery is still associated with a considerable morbidity. An important complication is ischemia of the flap –especially on the distal potion.^1^

Lots of clinical trials have tried to find ways to reduce distal flap necrosis. The delayed procedure can be very useful, but requires additional surgical interventions, takes a great deal of time, and can be assumed invasive.^2^ The factors involved in distal skin flap necrosis are either extrinsic or intrinsic. The extrinsic category consists of systemic or local causes such as malnutrition, hypotension, infection, compression, thrombosis, and kinking. The only significant intrinsic factor is arterial insufficiency.^3^

Preconditioning, systemic and topical agents and technical revisions are solutions mentioned in previous studies.^1^^-^^3^ Despite successes obtained, they had their own limitations. Safety and availability was an important item, especially in developing countries. So surgeons tried new trends with simple facilities. Vitamins are essential elements in our body managing cellular reactions like cell membrane transport and renewing tissues. Some vitamins act as antioxidants and reverse unwanted ischemic reactions. This study assessed the effect of topical vitamins A and E on improvement of distal ischemia of random skin flap in animal models. 

## MATERIALS AND METHODS

**Fig. 1:**
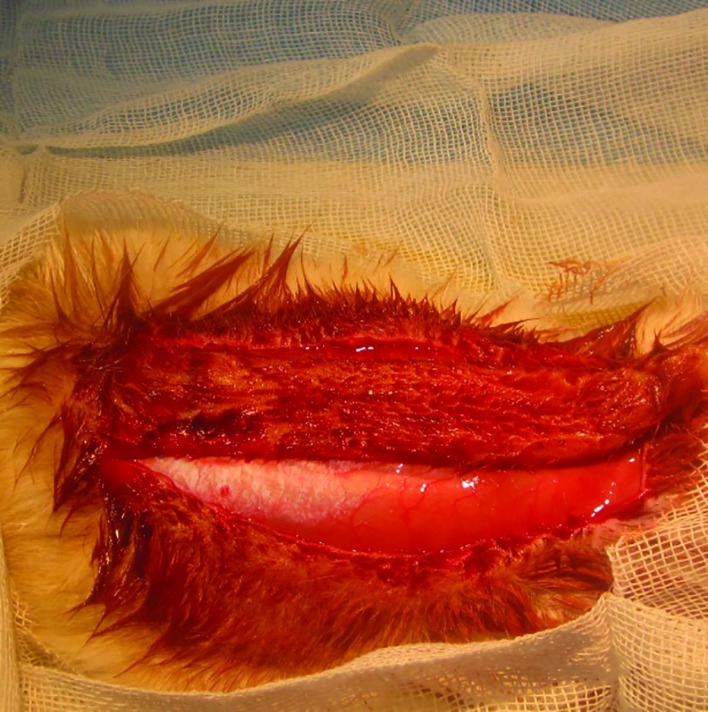
Caudally based random skin flap in 2x9 cm

A number of 24 white-albino male rats weighing 280-350 g living in animal lab were housed separately under the same conditions. They were divided randomly into two equal groups as case and control. All animals were anesthetized using 30-40 mg/kg sodium thiopental intra-peritoneally. They received 40 mg/kg keflin intraperitoneally too. Their backs were shaved and a 2x9 cm random caudally based pedicle dorsal flap was prepared according to an adaptation by McFarlane and colleagues (Figure 1).^4^

The flaps contained skin and panniculuscarnosus tissue. Sacral pedicles were saved at the flap base. No extra effort was undertaken for hemostasis, and the free edges of the flap were loosely sutured in place with 4-0 nylon. To avoid the animals from harming themselves, dressings were used on the operative site. Animals received their treatments every day according to their groups by a different researcher as drug A and B (mixture of vitamins A and E in vaseline cream and pure vaseline cream as placebo, respectively. 

**Fig. 2:**
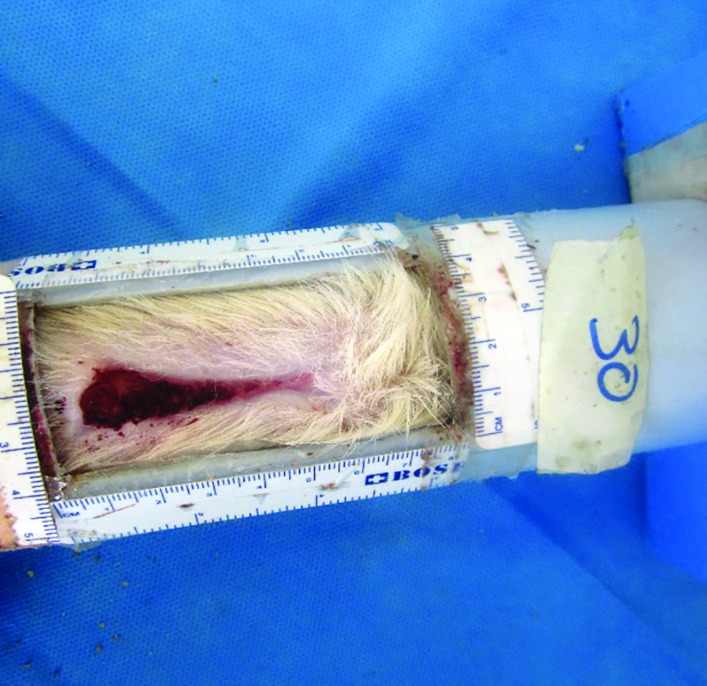
Measurement of necrotic area 10 days after flap elevation

The topical agents were rubbed on the surface of the flaps. Ten days after surgery, the animals were examined by the third examiner and the amount of necrotic segment of the flap was measured with metric ruler in contrast to healthy segment (Figure 2). The results were presented as percentages of skin necrosis area (mean±standard deviation). The difference in the mean percentage of flap necrosis between the two groups was analyzed with statistical package for social sciences (SPSS) software (version 16, Chicago, IL, USA). A p value less than 0.05 was considered statistically significant.

## RESULTS

**Fig. 3:**
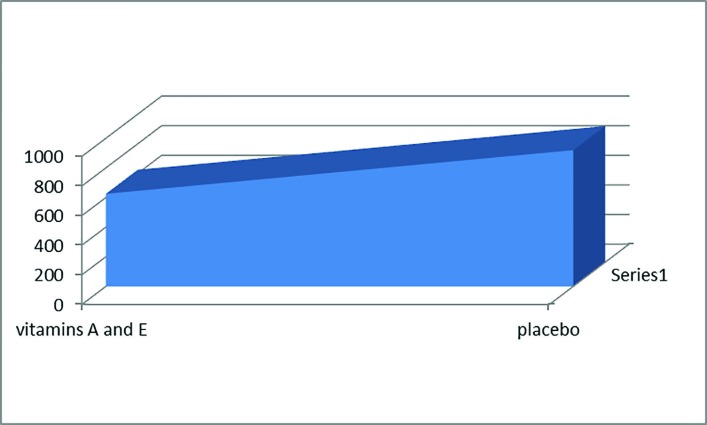
Mean necrotic area of flaps

Among 25 animals underwent surgery, one rat died after surgery. So 24 rats finished the study. The mean value of necrosis in the flaps were 625±189.56 mm^2 ^and 920.00±247.31 mm^2 ^in the treatment and control groups, respectively (Figure 3). In the control group (n=12), the mean area of the flaps was 1800±20 mm^2^, and the mean necrotic area of flap was 920±247 mm^2^ (51%). In the treatment group (n=12), the mean area of the flap was 1800±20 mm^2^, and the mean necrotic area of flap was 625±189.56 mm^2^ (34%). Using the Student’s t test, the difference between results of both groups showed that vaseline plus vitamins A and E affected skin flap area survival (*p*=0.085).

## DISCUSSION

Random pattern skin flaps are frequent reconstructive procedures, though, skin flap necrosis is of concern. Many clients encountered arterial or venous insufficiencies as the cause of flap failure for many years. Surgical delay and systemic pharmaceuticals are known solutions with their own costs. Harder and colleagues compared different preconditioning tools in improvement of flap survival.^5^ Machado *et al.* compared effects of transcutaneous electrical nerve stimulation on wound healing.^6^ Comparing topical phenytoin and capsaicin, Koskaland and colleagues found better results among necrotic flaps of rats with capsaicin.^7^ However, Kjartansson *et al.* had reported that survival of experimental critical flaps in rats after sensory denervation with capsaicin decreased.^8^ Carnitine as an endogenous cofactor necessary for cellular reactions, has been considered by scientists as a kind of life boat in ischemic conditions.^9^^,^^10^

Vitamins are among cofactors used daily and can be used as medicines in ischemic conditions.^11^^, ^^12^ The effect of vitamin A deficiency on wound healing is mostly cited and variable types of vitamin A supplement on improving healing have been accepted, and anti-oxidant effects of vitamin E could help cells in problematic conditions. Hayden *et al.* (1987) supposed free radical scavengers such as glutathione and vitamins A, C, and E can save endangered ischemic flaps.^13^ Bilgin-Karabulut *et al.* (2001) demonstrated prophylactic synergistic (but not solely) effects of these vitamins on venous congestion of flaps.^14^

Both studies notified positive effects of vitamins on flaps and we took the key point and established our study on survey by available topical agents and based on the above mentioned studies and we found topical agents to have advantage on others according to cost, feasibility, easy to use and being reproducible. Our results showed improvement in percentage of flap survival in animals treated with topical vitamins of A and E. This can be due to their role as free oxygen radical scavenger that deletes hazardous factors from reconstructed tissues. 

In this study, influencing factors were controlled to lower unwanted sequel. Further studies are suggested among more than one topical agent to obtain and make a mixture including effective drugs. Random fasciocutaneous flaps can be assumed as essential saving boats in reconstructive surgery with probable ischemic threatening. We consider topical vitamins as available inexpensive and easily used materials to diminish ischemic consequences and recommend more studies with pathological evidences on cellular improvement to introduce mixed pharmaceutical agents. 
